# Contribution of Organic Anion Transporter 3 in Delayed Elimination of Methotrexate by Concomitant Administration of Febuxostat

**DOI:** 10.1002/bdd.70014

**Published:** 2025-08-25

**Authors:** Kenji Ikemura, Sakura Kobayashi, Danni Wang, Masahiro Okuda

**Affiliations:** ^1^ Department of Pharmacy The University of Osaka Hospital Osaka Japan; ^2^ Department of Hospital Pharmacy Graduate School of Medicine The University of Osaka Osaka Japan; ^3^ Department of Hospital Pharmacy School of Pharmaceutical Sciences The University of Osaka Osaka Japan

**Keywords:** drug interaction, febuxostat, methotrexate, organic anion transporter 3

## Abstract

Methotrexate is an antifolate agent used for the treatment of various malignancies and is mainly secreted via human organic anion transporter 3 (hOAT3) in the proximal tubular cells. Coadministration of the xanthine oxidase inhibitor, febuxostat, in patients receiving methotrexate has been reported to be associated with an elevated risk of hematological toxicity and increased plasma methotrexate levels. Because febuxostat has an inhibitory effect against hOAT3, it may inhibit renal elimination of methotrexate via hOAT3. However, the drug interaction between methotrexate and febuxostat via hOAT3 remains to be clarified. In the present study, we investigated the effect of febuxostat on pharmacokinetics of methotrexate in rats and drug interaction between methotrexate and febuxostat using hOAT3‐expressing cultured cells. In the pharmacokinetics study using rats, concomitant administration of febuxostat significantly increased plasma concentration of methotrexate and prolonged its half‐life. In vitro studies showed that febuxostat inhibited hOAT3‐mediated transport of methotrexate in a concentration‐dependent manner. Dixon plot indicated that inhibitory constant value of febuxostat against methotrexate transport via hOAT3 was 0.63 ± 0.01 μM. Moreover, the inhibitory effect of febuxostat was of noncompetitive type. Taken together, these results suggest that concomitant administration of febuxostat delayed elimination of methotrexate, at least in part, by noncompetitive inhibition of hOAT3‐mediated methotrexate transport at clinical concentrations. The findings of this study provide novel information on drug interactions associated with febuxostat.

## Graphical abstract

Coadministration of the xanthine oxidase inhibitor, febuxostat, delayed elimination of methotrexate, at least in part, by noncompetitive inhibition of human organic anion transporter 3‐mediated methotrexate transport at clinical concentrations. The findings of this study provide novel information on drug interactions associated with febuxostat.

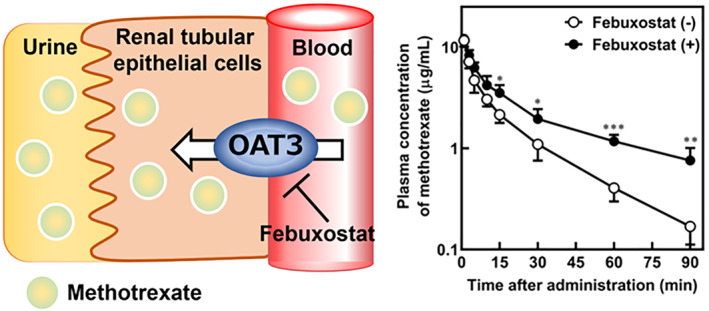

## 1 Introduction

Methotrexate, an antifolate agent, inhibits dihydrofolate reductase. High‐dose methotrexate, defined as a dose of ≥ 500 mg/m^2^, is widely used for the treatment of various malignant diseases, including leukemia, lymphoma, acute lymphoblastic, and osteosarcoma (Howard et al. 2016). Since delayed elimination of methotrexate can lead to the development of severe adverse effects, such as myelosuppression, nephrotoxicity, and hepatotoxicity (Faria et al. 2023), therapeutic drug monitoring of methotrexate is crucial for avoiding severe toxicities in patients treated with high‐dose methotrexate.

Methotrexate is mainly eliminated by the kidneys, and approximately 95% of the drug is excreted in urine as unchanged drug within 30 h of administration (Bleyer 1978). Although methotrexate undergoes reabsorption in proximal tubular cells, this reabsorption becomes saturated during high‐dose methotrexate therapy, resulting in nonlinear pharmacokinetics (Hendel and Nyfors 1984). Thus, renal tubular secretion significantly contributes to the renal clearance of methotrexate during high‐dose methotrexate therapy.

Human organic anion transporters, hOAT1 (*SLC22A6*) and hOAT3 (*SLC22A8*), play a crucial role in basolateral uptake of methotrexate in proximal tubular cells (Jafari et al. 2023). Furthermore, ATP‐binding cassette transporters, including breast cancer resistance protein (BCRP/*ABCG2*) and multidrug resistance‐associated protein 2 (MRP/*ABCC2*), are implicated in the excretion of methotrexate into urine through the brush‐border membrane of proximal tubules (Jafari et al. 2023). Previous reports have demonstrated that the elimination of methotrexate could be delayed due to concomitant administration of probenecid (Aherne et al. 1978), proton pump inhibitors (Chioukh et al. 2014; Narumi et al. 2017; Ueda et al. 2020), and nonsteroidal anti‐inflammatory drugs (Maeda et al. 2008), which are well‐investigated as inhibitors of hOAT3. Therefore, these findings suggest that particular care should be taken during coadministration of inhibitors and/or substrates of hOAT3 in patients receiving methotrexate.

Febuxostat is a nonpurine selective xanthine oxidase inhibitor that effectively prevents hyperuricemia accompanied by tumor lysis syndrome during cancer chemotherapy using high‐dose methotrexate (Spina et al. 2015). Analysis of the Japanese Adverse Drug Event Report (JADER) database demonstrated that concomitant administration of febuxostat with methotrexate enhanced the risk of hematological toxicity in patients receiving intravenous methotrexate (Mitsuboshi et al. 2022). In our previous study, concomitant febuxostat administration significantly increased serum methotrexate concentrations at 48 and 72 h after administration of methotrexate in patients treated with high‐dose methotrexate (Ikemura et al. 2019). As the risk of hematologic toxicity generally depends on the serum concentration of methotrexate, it is likely that the delayed elimination of methotrexate due to concomitant febuxostat administration contributes to an elevated risk of hematologic toxicity. Interestingly, recent studies reported that febuxostat potently inhibited hOAT3‐mediated transport of hOAT3 substrates at clinical concentrations (Ni et al. 2020; Tang et al. 2022). Taking these findings into consideration, we hypothesize that coadministration of febuxostat may exacerbate the hematological toxicity of methotrexate by inhibiting the renal elimination of methotrexate via hOAT3. However, the drug interaction via hOAT3 between methotrexate and febuxostat remains to be fully clarified. In addition, information regarding this interaction is not provided in the package insert of febuxostat (Feburic Tablet, Teijin Pharma Limited, Japan).

In the present study, we investigated the effect of febuxostat on pharmacokinetics of methotrexate in rats as well as drug interaction between methotrexate and febuxostat using hOAT3‐expressing cultured cells.

## 2 Materials and Methods

### 2.1 Materials

Febuxostat and 6‐carboxyfluorescein (6‐CF) were sourced from Tokyo Chemical Industry Co. Ltd. (Tokyo, Japan). Probenecid and methotrexate were acquired from FUJIFILM WAKO Pure Chemical (Osaka, Japan). All other chemicals utilized were of the highest available purity.

### 2.2 Pharmacokinetics Study of Methotrexate After Intravenous Administration of Methotrexate Along With Febuxostat in Rats

Nine‐week‐old male Wistar rats (SLC Japan Co., Shizuoka, Japan) were used for the pharmacokinetics study. All animal procedures were approved by the Animal Experiments Committee of The University of Osaka (No. 03‐018‐000) and conducted in accordance with the Japanese Law for the Protection and Management of Animals, the Standards Relating to the Care and Management of Laboratory Animals and Relief of Pain, and other relevant regulations and guidelines for animal experimentation. The rats were anesthetized with an intraperitoneal injection of a mixture of medetomidine, midazolam, and butorphanol at doses of 0.38, 2.0, and 2.5 mg/kg, respectively. Polyethylene catheters were implanted into the femoral vein and femoral artery to administer the drug and facilitate frequent blood collection. Subsequently, the rats received intravenous injection (i.v.) of methotrexate (2 mg/kg) with and without febuxostat (1 mg/kg, i.v.) through the femoral vein. Blood samples were obtained from the femoral artery at 0, 1, 3, 5, 10, 15, 30, 60, and 90 min after methotrexate administration. Plasma concentrations of methotrexate were determined by ultra‐performance liquid chromatography equipped with tandem mass spectrometry (UPLC‐MS/MS). The area under the plasma concentration–time curve from 0 to 90 min (AUC_0–90 min_) was calculated using the trapezoidal rule. Moreover, the systemic clearance (CL_tot_), elimination rate constant from the central compartment (K_el_), distribution volume of the central compartment (V_d_), and half‐life (T_1/2_) were calculated according to the procedures for 2‐compartmental analysis.

### 2.3 Cell Culture

The hOAT3‐expressing human embryonic kidney cell line HEK293 (HEK‐hOAT3) and mock‐transfectants obtained by transfecting pBKCMV vector into HEK293 cells (HEK‐pBK) were generously provided by Dr. Atsushi Yonezawa (Department of Pharmacy, Kyoto University Hospital, Japan). These cells were cultured in complete medium consisting of Medium 199 (Life Technologies, Carlsbad, CA) supplemented with 10% fetal bovine serum containing G418 (0.5 mg/mL; FUJIFILM WAKO Pure Chemical) and were used between passage numbers 95 and 115. These cells were maintained at 37°C under 5% CO_2_ in a humidified atmosphere.

### 2.4 Uptake Study of 6‐CF and Methotrexate in HEK‐pBK and HEK‐hOAT3 Cells

The cells (12 × 10^5^ cells/dish) were seeded in 3.5 cm dishes with culture medium in the absence of G418. After 48 h of culture, cell monolayers were utilized for uptake study. The cellular uptake of 6‐CF (a well‐established substrate of hOAT3) or methotrexate was determined using monolayer cultures of HEK‐pBK and HEK‐hOAT3 cells. The composition of the incubation medium was as follows: 145 mM NaCl, 3 mM KCl, 1 mM CaCl_2_, 0.5 mM MgCl_2_, 5 mM D‐glucose, and 5 mM HEPES (pH 7.4). After preincubation with the incubation medium for 10 min at 37°C, the cells were incubated with 5 μM 6‐CF for 2 min or with 10 μM methotrexate for a specified duration at 37°C. For inhibition experiments in HEK‐hOAT3 cells, the cells were incubated with 5 μM 6‐CF for 2 min in the absence or presence of 100 μM probenecid (a typical inhibitor of hOAT3) or with 10 μM methotrexate for 2 min in the absence or presence of various concentrations of febuxostat. To evaluate the accumulation of methotrexate into the cells, methotrexate was eluted with 0.5 mL of 50% methanol and then subjected to UPLC‐MS/MS. To assess intracellular 6‐CF accumulation, the cells were solubilized in 1 N NaOH and fluorescence was measured using a fluorescence spectrophotometer (SH‐9000lab, CORONA, Ibaraki, Japan) at 495 nm excitation/517 nm emission. The protein content of the solubilized cells was measured using a BCA protein assay kit (Thermo Fisher Scientific, Waltham, MA).

### 2.5 Determination of Methotrexate in Plasma and Cells

UPLC‐MS/MS was employed for determination of methotrexate in plasma and cells. The UPLC‐MS/MS system was applied with the ACQUITY HPLC H‐class/ACQUITY TQD system with electrospray ionization (Waters, Milford, MA). 10 μL of 100 ng/mL pemetrexed, utilized as an internal standard (IS), was added to the samples (100 μL). The samples (10 μL) were then subjected to UPLC‐MS/MS. LC separations were performed on an InterSustainSwift C18 HP column (2.1 × 150 mm, 3 μm, GL Sciences, Tokyo, Japan) maintained at 40°C with a flow rate of 0.2 mL/min. Solvent A was water with 0.1% formic acid and solvent B was acetonitrile with 0.1% formic acid. The entire LC gradient was 16 min. Mobile phase B was initially at 10%, ramped to 95% from 1 to 12 min, and then back to 10% from 12 to 16 min. Methotrexate and IS were detected using the multiple reaction monitoring mode. MS/MS conditions involved cone voltages and collision energies of 40 V/40 eV (methotrexate) and 40 V/20 eV (IS) in positive mode, respectively. MS/MS monitoring ions were as follows: methotrexate (*m/z* 455.26 [M + H]^+^ → *m/z* 175.26) and IS (*m/z* 428.28 [M + H]^+^ → *m/z* 281.30). The desolvation temperature was 350°C, cone gas flow was 50 L/h, and desolvation gas flow was 600 L/h. All UPLC‐MS/MS data were collected and processed employing the Masslynx 4.1 software (Waters).

### 2.6 Kinetic Analyses

Kinetic analyses were performed using GraphPad Prism version 8.4.3 (GraphPad Software Inc. San Diego, CA). The apparent Michaelis–Menten constant (*K*_m_) and maximal velocity (*V*_max_) values were calculated using the Michaelis–Menten equation: *V* = *V*_max_ · *S*/(*K*_m_ + *S*), where *V* is the transport velocity, *S* is the concentration of methotrexate, *V*_max_ is the maximal velocity, and *K*_m_ is Michaelis–Menten constant employing nonlinear regression analysis. The apparent IC_50_ values were calculated from the inhibition plots according to the equation: *V* = *V*_bottom_ + (*V*_top_ − *V*_bottom_)/[1 + (log *I*/IC_50_)^*n*^] using nonlinear least square regression analysis, where *V* is the transport velocity, *V*_bottom_ is transport velocity at the highest concentration of inhibitor, *V*_top_ is transport velocity without inhibitor, *I* is concentration of the inhibitor, and *n* is the Hill coefficient.

### 2.7 Statistical Analyses

In vitro and in vivo experimental data are expressed as mean ± standard error (S.E.) and mean ± standard deviation (S.D.), respectively. Statistical comparisons between two groups were performed using unpaired *t*‐test. Statistical analyses were performed using GraphPad Prism version 8.4.3. A two‐tailed *p*‐value < 0.05 was considered statistically significant.

## 3 Results

### 3.1 Pharmacokinetic Study of Methotrexate After Intravenous Administration of Methotrexate With Febuxostat in Rats

**FIGURE 1:**
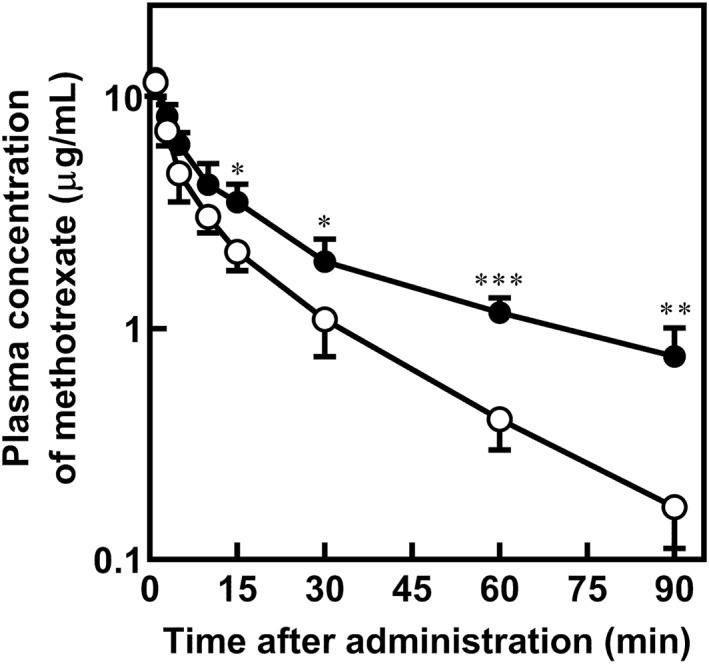
Plasma concentration of methotrexate after intravenous administration of methotrexate (2 mg/kg) without (open circles) or with (closed circles) febuxostat (1 mg/kg) in rats. Each point represents the mean ± S.D. of four rats. *p < 0.05, **p < 0.01, and ***p < 0.001 compared to rats treated without febuxostat.

To verify whether the plasma concentration of methotrexate is affected by concomitant administration of febuxostat, we conducted a pharmacokinetic study in rats after intravenous administration of methotrexate. According to the review report of febuxostat (Feburic Tablet), plasma concentration of febuxostat was approximately 24.1 μM at 5 min after intravenous administration of febuxostat (1 mg/kg) in rats. Since the protein binding of febuxostat is 98.4% in rats (Yu et al. 2023), the estimated unbound plasma concentration of febuxostat is approximately 0.40 μM, which is comparable to clinical concentration (0.12–0.30 μM). Therefore, the dose of febuxostat was set at 1 mg/kg. Plasma concentration–time profiles of methotrexate following administration of methotrexate (2 mg/kg) with or without febuxostat (1 mg/kg) are shown in Figure 1. The plasma concentration of methotrexate was significantly increased at 15, 30, 60, and 90 min by concomitant febuxostat administration. The pharmacokinetic parameters of methotrexate are summarized in Table 1. The concomitant use of febuxostat significantly increased the AUC_0–90 min_ and significantly decreased the CL_tot_ of methotrexate. Moreover, decreased K_el_ and prolonged T_1/2_ of methotrexate were observed because of administration of febuxostat, whereas there was no significant difference in the V_d_ value of methotrexate between rats treated with and without febuxostat.

**TABLE 1:** Pharmacokinetic parameters of methotrexate after intravenous administration of methotrexate with and without febuxostat in rats.

Parameters	Febuxostat		*p* value
	(−)	(+)	
AUC_0–90 min_ (μg·min/mL)	130 ± 25	261 ± 59	0.007
CL_tot_ (mL/min/kg)	15.92 ± 3.56	8.02 ± 2.03	0.008
K_el_ (min^−1^)	0.11 ± 0.03	0.05 ± 0.02	0.016
V_d_ (mL/kg)	378 ± 61	433 ± 88	0.208
T_1/2_ (min)	6.4 ± 1.4	14.0 ± 4.6	0.019

*Note:* Results represent the mean ± S.D. (*n* = 4). AUC_0–90 min_, area under the plasma concentration from 0 to 90 min after methotrexate administration. The AUC was calculated using the trapezoidal rule. Statistical analyses were performed using unpaired *t*‐test.

Abbreviations: CL_tot_, systemic clearance; K_el_, elimination rate constant from the central compartment; T_1/2_, half‐life; V_d_, distribution volume of the central compartment.

### 3.2 Uptake of 6‐CF in HEK‐pBK and HEK‐hOAT3 Cells

To confirm the activity of hOAT3 in HEK‐hOAT3 cells, we conducted an uptake study of 5 μM 6‐CF (Figure S1). The uptake of 6‐CF in HEK‐hOAT3 cells was approximately 17.4‐fold higher than in HEK‐pBK cells, the corresponding controls (Figure S1A). The uptake of 6‐CF in HEK‐hOAT3 cells was potently inhibited by co‐incubation with probenecid (Figure S1B). These results confirmed the activity of hOAT3 in HEK‐hOAT3 cells.

### 3.3 Uptake of Methotrexate in HEK‐pBK and HEK‐hOAT3 Cells

**FIGURE 2:**
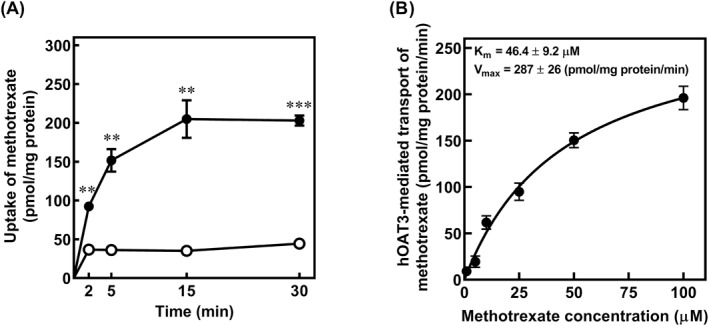
(A) HEK‐hOAT3 (closed circles) or HEK‐pBK (open circles) cells were incubated with methotrexate (10 μM, pH 7.4) for specified durations (2, 5, 10, and 15 min) at 37°C. (B) Concentration‐dependent uptake of hOAT3‐mediated methotrexate transport. The cells were incubated at 37°C for 2 min with various concentrations (1, 5, 10, 25, 50, and 100 μM) of methotrexate (pH 7.4). The uptake mediated by hOAT3 was obtained by subtracting the uptake in HEK‐pBK cells from that in HEK‐hOAT3 cells. Each point represents mean ± S.E. of three separate experiments using three monolayers. When the standard errors of the means are small, they are contained within the symbols. **p < 0.01 and ***p < 0.001 compared to HEK‐pBK cells.

Uptake of 10 μM methotrexate in HEK‐pBK and HEK‐hOAT3 cells was evaluated. As shown in Figure 2A, the uptake of methotrexate in HEK‐hOAT3 cells increased in a time‐dependent manner and reached an equilibrium state after 15 min. Moreover, the uptake of methotrexate in HEK‐hOAT3 cells was significantly higher than that in HEK‐pBK cells at all time points. To examine the characteristics of methotrexate transport via hOAT3, concentration‐dependent uptake studies for 2 min were conducted. Figure 2B shows the concentration‐dependent uptake of methotrexate via hOAT3 by subtracting the uptake in HEK‐pBK cells from that in HEK‐hOAT3 cells. The uptake of hOAT3‐mediated transport of methotrexate was saturated at high concentrations. From the results shown in Figure 2B, the apparent *K*_m_ and *V*_max_ values of hOAT3‐mediated uptake of methotrexate were 46.4 ± 9.2 μM and 287 ± 26 pmol/mg protein/min, respectively.

### 3.4 Inhibition of hOAT3‐Mediated Transport of Methotrexate by Febuxostat

**FIGURE 3:**
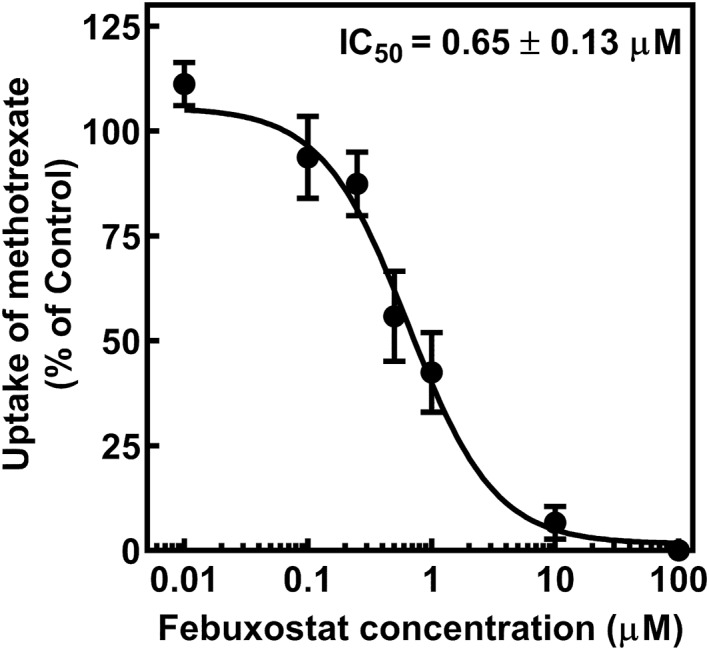
Inhibition of methotrexate uptake by febuxostat in HEK‐hOAT3 cells. HEK‐hOAT3 cells were incubated at 37°C for 2 min with 10 μM methotrexate (pH 7.4) in the absence or presence of various concentrations of febuxostat. Each point represents mean ± S.E. of three separate experiments using three monolayers. When the standard errors of the means are small, they are contained within the symbols. The apparent IC_50_ values were calculated by fitting the data to a sigmoidal dose–response regression curve.

To assess whether febuxostat inhibit hOAT3‐mediated transport of methotrexate, cellular uptake of methotrexate (10 μM) was measured for 2 min in the absence or presence of various concentrations of febuxostat (Figure 3). Febuxostat inhibited hOAT3‐mediated transport of methotrexate in a concentration‐dependent manner. As shown in Figure 3, the apparent IC_50_ value of febuxostat was 0.65 ± 0.13 μM.

### 3.5 Dixon Plot of the Inhibitory Effect of Febuxostat Against hOAT3‐Mediated Transport of Methotrexate

**FIGURE 4:**
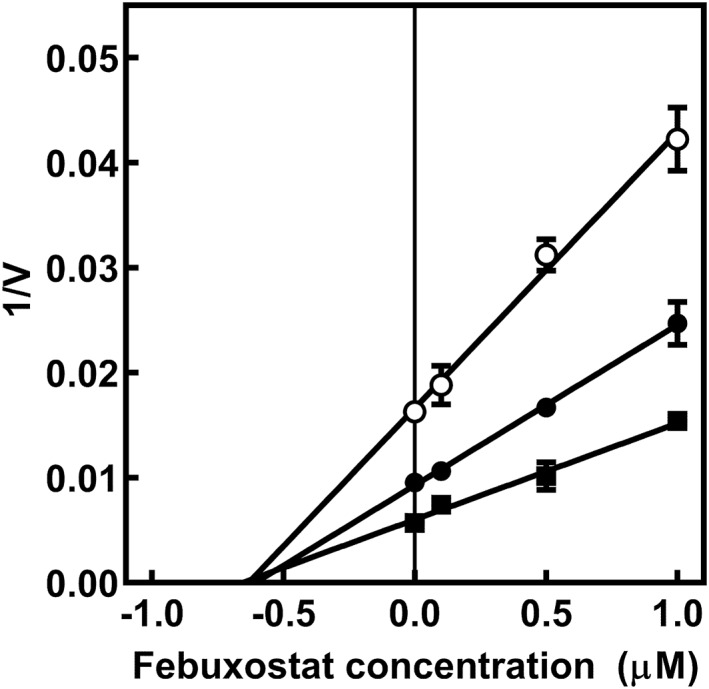
Dixon plot of the inhibition of methotrexate uptake by febuxostat in HEK‐hOAT3 cells. HEK‐hOAT3 cells were incubated at 37°C for 2 min with 10 μM (open circles), 25 μM (closed circles), and 50 μM (closed squares) of methotrexate (pH 7.4) in the absence or presence of febuxostat (0, 0.1, 0.5, and 1.0 μM). Each point represents mean ± S.E. of three separate experiments using three monolayers. When the standard errors of the means are small, they are contained within the symbols. V represents the uptake velocity (pmol/mg protein/min).

A Dixon plot was constructed to clarify the type of inhibition of febuxostat against hOAT3‐mediated transport of methotrexate (Figure 4). Cellular uptake of methotrexate (10, 25, and 50 μM) was measured for 2 min in the absence and presence of febuxostat (0.1, 0.5, and 1.0 μM). The Dixon plot clearly indicated that the inhibitory type of febuxostat against hOAT3‐mediated transport of methotrexate was noncompetitive; the inhibitory constant (Ki) value was 0.63 ± 0.01 μM.

## 4 Discussion

Drug interaction via hOAT3 between methotrexate and febuxostat remains to be clarified. Our study demonstrated for the first time that concomitant administration of febuxostat delayed elimination of methotrexate, at least in part, by noncompetitive inhibition hOAT3‐mediated transport of methotrexate.

In a retrospective study of patients receiving high‐dose methotrexate therapy (Ikemura et al. 2019), concomitant febuxostat administration significantly increased the serum methotrexate concentration at 48 and 72 h after administration of methotrexate. Thus, we evaluated the effect of febuxostat at clinical doses on the pharmacokinetics of methotrexate after intravenous administration of methotrexate in rats (Figure 1). As shown in Figure 1, plasma concentration of methotrexate was drastically elevated by concomitant administration of febuxostat. In the pharmacokinetic analysis (Table 1), decreased K_el_ and prolonged T_1/2_ of methotrexate were observed by administration of febuxostat. Thus, these findings suggest that coadministration of febuxostat delays elimination of methotrexate in humans.

Methotrexate is transported via rat OAT1 (rOAT1) and rOAT3, exhibiting higher affinity for rOAT3 than rOAT1 (Nozaki et al. 2004), consistent with findings in humans (Kurata et al. 2014). Uptake study using rat kidney slices demonstrated that rOAT3 is responsible for approximately 50% of renal basolateral uptake of methotrexate, and rOAT1 accounts for only a limited contribution (Nozaki et al. 2004). Furthermore, it has been reported that concomitant administration of OAT3 inhibitors, such as proton pump inhibitors, delays methotrexate excretion in rats (Ueda et al. 2020). In Figure 1, the prolonged elimination of methotrexate by febuxostat could be primarily attributed to the inhibition of its tubular secretion via rOAT3 in rats.

Various drug transporters, including OATs, BCRP, and MRP2, are known to be responsible for renal excretion of methotrexate (Jafari et al. 2023). Because febuxostat is known to have a potent inhibitory effect on BCRP activity (Miyata et al. 2016), it can be speculated that the increased risk of hematological toxicity is attributable to BCRP‐mediated drug interaction. However, the inhibition of BCRP did not affect urinary excretion of methotrexate after intravenous methotrexate administration in mice (Breedveld et al. 2004). In addition, *ABCG2* mRNA is negligibly expressed in the human kidney (Doyle et al. 1998). In contrast, some studies have demonstrated that the concomitant administration of OAT3 inhibitors significantly impairs the renal elimination of methotrexate (Aherne et al. 1978; Chioukh et al. 2014; Narumi et al. 2017). Thus, it is assumed that OAT3 rather than BCRP plays a crucial role in the urinary excretion of methotrexate in humans.

Recently, Tang et al. (2022) reported that febuxostat potently inhibited the transport of estron‐3‐sulfate and rivaroxaban in hOAT3‐expressing HEK293 cell. We hypothesize that coadministration of febuxostat may delay elimination of methotrexate by inhibiting renal elimination of methotrexate via hOAT3. However, hOAT3‐mediated drug interaction between methotrexate and febuxostat remained to be clarified. As shown in Figure 3, the inhibition of hOAT3‐mediated transport of methotrexate was confirmed by co‐incubation of febuxostat in a concentration‐dependent manner. The Dixon plot clearly indicated that the inhibitory type of febuxostat of hOAT3 was noncompetitive and Ki value of febuxostat was 0.63 ± 0.01 μM (Figure 4). As Tang et al. (2022) reported that the Ki value of febuxostat against hOAT3 was 0.55 ± 0.15 μM, our findings are consistent with the results of Tang et al. (2022). Thus, the present study is the first to report hOAT3‐mediated drug interaction between methotrexate and febuxostat.

Guidelines regarding transporter‐mediated drug interactions published by the U.S. FDA in 2020 suggest that a ratio of unbound *C*_max_ to IC_50_ or Ki value ≥ 0.1 indicates that clinical drug interaction should be evaluated. When 40–120 mg of febuxostat was orally administered in human, the *C*_max_ of febuxostat was approximately 5.3–13.5 μM (Zhang et al. 2014). Because the protein binding of febuxostat is 97.8%, the unbound *C*_max_ of febuxostat was estimated to be 0.12–0.30 μM. In our study, the ratio of unbound *C*_max_ to Ki value of febuxostat was 0.19–0.48 (≥ 0.1), implying that coadministration of febuxostat and methotrexate could lead to clinical drug interaction.

Because Zou et al. (2021) reported that various drug metabolites serve as substrates and/or inhibitors of hOAT1 and hOAT3, these may affect both therapeutic outcomes and adverse drug reactions through the inhibition of renal drug transporters. Therefore, careful attention should be paid to drug interactions involving drug metabolites. Febuxostat undergoes metabolism through uridine diphosphate‐glucuronosyltransferase, resulting in the formation of a major metabolite, febuxostat acyl‐β‐D‐glucuronide (Kamel et al. 2017). Febuxostat acyl‐β‐D‐glucuronide is mainly eliminated by the kidneys (Kamel et al. 2017). In addition, secretion of some glucuronide metabolites into proximal tubules can be inhibited by probenecid, a potent hOAT3 inhibitor (Meffin et al. 1983; Veenendaal et al. 1981). Consequently, drug interaction via hOAT3 between methotrexate and febuxostat acyl‐β‐D‐glucuronide may potentially occur. Tang et al. (2022) reported that febuxostat acyl‐β‐D‐glucuronide also has an inhibitory effect on hOAT3. In an uptake study using HEK‐hOAT3 cells with estron‐3‐sulfate (a typical hOAT3 substrate), Ki value of the febuxostat acyl‐β‐D‐glucuronide against hOAT3 was 6.11 μM and its inhibitory type was competitive (Tang et al. 2022). Thus, febuxostat as a parent drug exhibited a more potent inhibitory effect against hOAT3 than febuxostat acyl‐β‐D‐glucuronide, suggesting that febuxostat exerts a greater influence on the renal elimination of methotrexate than its glucuronide metabolite. However, we could not evaluate the clinical drug interaction between methotrexate and febuxostat acyl‐β‐D‐glucuronide because little information is available on the plasma concertation of febuxostat acyl‐β‐D‐glucuronide in humans treated with febuxostat. Thus, further studies are required to assess the effect of febuxostat acyl‐β‐D‐glucuronide on the pharmacokinetics of methotrexate.

## 5 Conclusion

In conclusion, our study is the first to demonstrate that concomitant administration of febuxostat delayed elimination of methotrexate, at least in part, by noncompetitive inhibition of hOAT3‐mediated methotrexate transport at clinical concentrations. The present findings provide novel information on drug interactions associated with febuxostat.

## Conflicts of Interest

The authors declare no conflicts of interest.

## Supporting information


**Figure S1:** (A) Uptake of 6‐carboxyfluorescein (6‐CF) in HEK‐pBK and HEK‐hOAT3 cells. (B) Effect of probenecid on the uptake of 6‐CF in HEK‐hOAT3 cells. The cells were incubated for 2 min at 37°C with 5 µM 6‐CF (pH 7.4) in the absence or presence of probenecid (100 µM). Each column represents the means ± S.E. of three separate experiments using three monolayers. ***p < 0.001 compared with HEK‐pBK cells. ^###^ *p* < 0.01 compared with probenecid (–).

## Data Availability

All data generated or analyzed during this study are included in this published article and Figure S1.
