# Supplementary material for: Contribution of Organic Anion Transporter 3 in Delayed Elimination of Methotrexate by Concomitant Administration of Febuxostat
Source: Biopharm Drug Dispos. 2025 Aug 25;46(4):165–71. doi: 10.1002/bdd.70014 (PMC12417622; doi:10.1002/bdd.70014)
Supplement: Supplementary file 1 — Figure S1: (A) Uptake of 6‐carboxyfluorescein (6‐CF) in HEK‐pBK and HEK‐hOAT3 cells. (B) Effect of probenecid on the uptake of 6‐CF in HEK‐hOAT3 cells. The cells were incubated for 2 min at 37°C with 5 µM 6‐CF (pH 7.4) in the absence or presence of probenecid (100 µM). Each column represents the means ± S.E. of three separate experiments using three monolayers. ***p < 0.001 compared with HEK‐pBK cells. ### p < 0.01 compared with probenecid (–). [file BDD-46-165-s001.pdf]

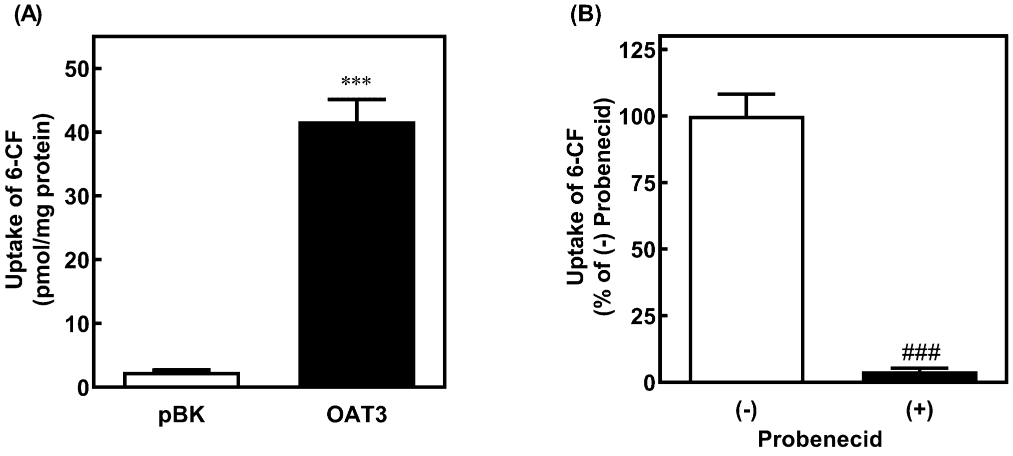


**Supplementary Figure 1.** (A) Uptake of 6-carboxyfluorescein (6-CF) in HEK-pBK and HEK-hOAT3 cells. (B) Effect of probenecid on the uptake of 6-CF in HEK-hOAT3 cells. The cells were incubated for 2 min at 37°C with 5 μM 6-CF (pH 7.4) in the absence or presence of probenecid (100 μM). Each column represents the means ± S.E. of three separate experiments using three monolayers. ***$p < 0.001$ compared with HEK-pBK cells. ###$p < 0.01$ compared with probenecid (–).
